# Lean body mass and the cardiorespiratory phenotype: An ethnic‐specific relationship in Hans Chinese women and men

**DOI:** 10.1002/jcsm.13464

**Published:** 2024-04-17

**Authors:** Meihan Guo, Candela Diaz‐Canestro, Nicola Riccardo Pugliese, Francesco Paneni, David Montero

**Affiliations:** ^1^ Faculty of Medicine Hong Kong University Hong Kong; ^2^ Department of Medicine, School of Clinical Medicine Hong Kong University Hong Kong; ^3^ Department of Clinical and Experimental Medicine University of Pisa Pisa Italy; ^4^ Center for Translational and Experimental Cardiology (CTEC), Zurich University Hospital University of Zurich Zurich Switzerland; ^5^ University Heart Center, University Hospital Zurich University of Zurich Zurich Switzerland

**Keywords:** Aerobic capacity, Body composition, Cardiovascular structure/function, Hans Chinese, Lean body mass, O_2_ extraction

## Abstract

**Background:**

Lean body mass (LBM) and the functional capacity of cardiovascular (CV) and respiratory systems constitute a female‐specific relationship in European–American individuals. Whether this recent finding be extrapolated to the world's largest ethnic group, that is, Hans Chinese (HC, a population characterized by low LBM), is unknown.

**Methods:**

Healthy HC adults (*n* = 144, 50% ♀) closely matched by sex, age and physical activity were included. Total and regional (leg, arm and trunk) LBM and body composition were measured via dual‐energy X‐ray absorptiometry. Cardiac structure, stiffness, central/peripheral haemodynamics and peak O_2_ consumption (VO_2peak_) were assessed via transthoracic echocardiography and pulmonary gas analyses at rest and during exercise up to peak effort. Regression analyses determined the sex‐specific relationship of LBM with cardiac and aerobic phenotypes.

**Results:**

Total and regional LBM were lower and body fat percentage higher in women compared with men (*P* < 0.001). In both sexes, total LBM positively associated with left ventricular (LV) mass and peak volumes (*r* ≥ 0.33, *P* ≤ 0.005) and negatively with LV end‐systolic and central arterial stiffness (*r* ≥ −0.34, *P* ≤ 0.004). Total LBM strongly associated with VO_2peak_ (*r* ≥ 0.60, *P* < 0.001) and peak cardiac output (*r* ≥ 0.40, *P* < 0.001) in women and men. Among regional LBM, leg LBM prominently associated with the arterio‐venous O_2_ difference at peak exercise in both sexes (*r* ≥ 0.43, *P* < 0.001). Adjustment by adiposity or CV risk factors did not modify the results.

**Conclusions:**

LBM independently determines internal cardiac dimensions, ventricular mass, distensibility and the capacity to deliver and consume O_2_ in HC adults irrespective of sex.

## Introduction

In an old‐fashioned physiological essay from the 1950s, the physician–scientist Wilhelm von Döbeln fragmentarily hinted the following hypothesis: body composition, independently of body weight, could primarily determine the human capacity to deliver and consume O_2_.^1^ Two‐thirds of a century later, strong independent relationships were recently demonstrated between lean body mass (LBM), peak cardiac output (Q_peak_) and consumption (VO_2peak_) in healthy adults.^2^ Moreover, LBM robustly associated with key structural and functional attributes of the left ventricle (LV) and periphery of the cardiovascular (CV) system underpinning Q_peak_ and VO_2peak_,^3^ which represent two powerful predictors of CV and overall mortality.^4^, ^5^ Unexpectedly, LBM relationships were found in women, whereas not even the slightest trend emerged in men, despite both sexes being matched by age, physical activity and established CV risk factors.^2^, ^3^ Consequently, we surmised the notion that LBM and the CV system constitute a female‐specific relationship.^3^ Potential clinical implications promptly emerged as LBM might reasonably be an effective and tractable target to enhance CV health in the female population.^6^, ^7^

The question arose: why was LBM utterly dissociated from the cardiac and aerobic phenotype in men?^2^, ^3^ A variable *excess* of muscle mass, that is, the predominant component of LBM,^8^ was hypothesized to be the primary explanation.^2^, ^3^ It should be noted that prior studies included European‐American (EA) individuals,^2^, ^3^ who rank among the ethnic groups with greater and more variable LBM in the male population.^9^, ^10^ May the sex specificity of our notion not be present in other ethnicities? Namely, would LBM and the CV system be intrinsically coupled in women as well as men from the world's largest ethnicity, that is, Hans Chinese (HC)? The HC ethnicity encompasses 20% of the human population, currently surpassing 1.3 billion individuals characterized by substantially lower LBM compared with EA, irrespective of sex.^11^ Scientific and pragmatical (global health) grounds thus merged to pose the questions in the HC population.

The purpose of this study was to determine the relationship of LBM with CV structural and functional variables underpinning the systemic capacity for O_2_ delivery and consumption in healthy HC women and men. A comprehensive relationship between LBM, cardiac and aerobic capacities independent of adiposity and CV risk factors was hypothesized to be present in HC adults irrespective of sex.

## Methods

### Study design

The study was approved by the Institutional Review Board of the University of Hong Kong/Hospital Authority West Cluster (UW 21‐401 and conducted in accordance with the declaration of Helsinki. A total of 144 healthy HC women and men (18–78 years) matched by age and physical activity (Table 1) were recruited via online advertisements in the city of Hong Kong (China). Moderate‐to‐vigorous physical activity (MVPA, total and specific to aerobic (endurance) exercise) over participant's lifetime as well as during the last 3 months prior to the study, the latter comprising a detailed description of physical activities, were assessed at screening, as previously described.^12^, ^13^ Inclusion criteria comprised healthy status according to health/clinical questionnaires and resting echocardiography/ECG screening, absence of current medical symptoms and medication (any type), and no history of cardiac, pulmonary, haematological or neuromuscular conditions or diseases. Healthy adults were included as a disease‐ and medication‐free representative sample of the HC population. In addition, all participants had to present definite self‐identified ethnicity (HC) supported by legal name, country of birth, residential history, as well as the absence of ancestors of distinct ethnicity. The self‐identified ethnicity method has demonstrated a very high percentage of agreement (>90%) with genetic‐based measures of ancestry specific to HC.^14^, ^15^ A few individuals (*n* = 6) belonging to mixed‐ethnic backgrounds were excluded from the study.

**Table 1:** General characteristics of study subjects

	♀	♂
*n*	72	72
Age (years)	39.0 ± 18.2	40.1 ± 18.3
Height (cm)	161.0 ± 6.6	172.6 ± 7.0*
Weight (kg)	55.2 ± 7.9	67.6 ± 9.0*
BMI (kg·m^−2^)	21.3 ± 2.5	22.7 ± 2.6*
BSA (m^2^)	1.57 ± 0.13	1.80 ± 0.14*
SBP (mmHg)	112.3 ± 15.0	121.7 ± 12.2*
DBP (mmHg)	71.9 ± 9.2	76.4 ± 9.9*
MAP (mmHg)	85.4 ± 10.3	91.5 ± 10.0*
HR (b.p.m.)	61.4 ± 10.7	60.7 ± 10.1
Hb (g·dL^−1^)	12.6 ± 1.0	14.7 ± 1.2*
Hct (%)	38.8 ± 3.0	45.1 ± 3.5*
MVPA (h·week^−1^)	5.1 ± 3.8	5.7 ± 4.2
MVPA‐endurance (h·week^−1^)	4.4 ± 3.4	4.7 ± 3.9
VO_2peak_ (mL·min^−1^·kg^−1^)	33.0 ± 8.6	43.8 ± 11.1*
Body composition		
Total BMC (kg)	1.89 ± 0.31	2.47 ± 0.38*
Total LBM (kg)	37.9 ± 5.6	52.5 ± 6.8*
Total fat (kg)	16.5 ± 4.2	13.9 ± 4.2*
Total fat (%)	29.1 ± 5.3	19.9 ± 4.4*
Total area (cm^2^)	1827 ± 170	2,186 ± 197*
Leg LBM (kg)	12.7 ± 2.3	18.1 ± 2.6*
Leg fat (kg)	6.6 ± 1.6	4.8 ± 1.3*
Leg fat (%)	33.1 ± 6.0	20.1 ± 4.3*
Leg area (cm^2^)	661 ± 69	784 ± 85*
Arm LBM (kg)	3.2 ± 0.6	5.6 ± 1.0*
Arm fat (kg)	1.9 ± 0.5	1.5 ± 0.4*
Arm fat (%)	35.2 ± 6.9	20.6 ± 4.9*
Arm area (cm^2^)	341 ± 39	438 ± 42*
Trunk LBM (kg)	18.2 ± 2.7	24.3 ± 3.4*
Trunk fat (kg)	6.7 ± 2.3	5.9 ± 2.5
Trunk fat (%)	25.8 ± 6.2	18.7 ± 5.5*
Chest area (cm^2^)	300 ± 39	336 ± 46*
Cardiac structure and function at rest		
RA (mL)	20.9 ± 7.1	29.1 ± 9.3*
TA e′ (cm·s^−1^)	16.4 ± 3.6	15.0 ± 3.5*
TA a′ (cm·s^−1^)	13.5 ± 3.6	13.3 ± 3.5
TA S′ (cm·s^−1^)	13.5 ± 2.1	12.7 ± 2.2*
TAPSE (cm)	2.40 ± 0.47	2.42 ± 0.51
LA (mL)	23.8 ± 7.8	30.8 ± 8.9*
LV_mass_ (g)	93.4 ± 26.3	135.0 ± 33.9*
IVSd (cm)	0.80 ± 0.17	0.93 ± 0.16*
LVIDd (cm)	3.90 ± 0.42	4.45 ± 0.46*
LVPWd (cm)	0.83 ± 0.18	0.90 ± 0.15*
LVRWT	0.43 ± 0.13	0.41 ± 0.08
LV Ees (mmHg·mL^−1^)	5.85 ± 2.66	4.77 ± 1.94*
LV Ea (mmHg·mL^−1^)	1.23 ± 0.25	1.04 ± 0.23*
LV Ed (mL^−1^·10^2^)	6.11 ± 1.50	4.69 ± 1.24*
LVEDV (mL)	102.1 ± 16.7	135.3 ± 28.1*
LVESV (mL)	19.7 ± 6.1	26.0 ± 9.0*
LV SV (mL)	82.7 ± 13.6	109.2 ± 22.4*
LV MPI	0.38 ± 0.12	0.38 ± 0.14
Mitral E (cm·s^−1^)	77.7 ± 15.8	73.4 ± 15.1
Mitral A (cm·s^−1^)	49.9 ± 12.8	48.3 ± 11.9
Mitral E/A ratio	1.69 ± 0.62	1.61 ± 0.51
Septal e′ (cm·s^−1^)	16.0 ± 3.1	15.1 ± 2.8
Septal a′ (cm·s^−1^)	11.2 ± 3.2	11.3 ± 2.6
E/e′ ratio	6.45 ± 1.70	6.19 ± 1.45

Data are expressed as mean ± SD.

BMC, bone mineral content; BSA, body surface area; E/e′ ratio, ratio of peak blood flow velocity to tissue myocardial velocity in early diastole; HR, heart rate; IVSd, interventricular septum thickness at end‐diastole; LA, left atrium; LBM, lean body mass; LV Ea, left ventricular‐arterial elastance; LV Ed, left ventricular diastolic elastance; LVEDV, left ventricular end‐diastolic volume; LV Ees, left ventricular end‐systolic elastance; LVIDd, left ventricular internal diameter at end‐diastole; LV_mass_, left ventricular mass; LV MPI, left ventricular myocardial performance index; LVPWd, left ventricular posterior wall thickness at end‐diastole; LVRWT, left ventricular relative wall thickness; MAP, mean arterial pressure; Mitral E/A ratio, ratio of peak blood flow velocity in early diastole (mitral E wave) to peak blood flow velocity in late diastole due to atrial contraction (mitral A wave); MVPA, moderate‐to‐vigorous physical activity; MVPA‐endurance, moderate‐to‐vigorous physical activity comprising aerobic (endurance) exercise; RA, right atrium; Septal a′, septal tissue velocity in late diastole; Septal e′, septal tissue velocity in early diastole; SV, stroke volume; TA a′, tricuspid annular tissue velocity in late diastole; TA e′, tricuspid annular tissue velocity in early diastole; TAPSE, tricuspid annular plane systolic excursion; TA S′, tricuspid annular peak systolic tissue velocity; VO_2peak_; peak O_2_ consumption.

* *P* < 0.05, men versus women.

All individuals were instructed to avoid strenuous exercise, alcohol and caffeine as well as to record fluid intake and maintain their usual daily dietary habits from 24 h prior to their visit to the laboratory. The measurements were performed after a fasting period (≥4 h) to avoid postprandial haemodynamic alterations, in a quiet room with controlled temperature between 22 and 23°C. According to previous studies, the menstrual phase (if applicable) was noted but not fixed for testing as it does not influence the study outcomes.^2^, ^3^ Prior to the start of the measurements, informed oral and written consents were obtained from the participants.

### Measurements

#### Body composition

Body composition was determined via dual‐energy X‐ray absorptiometry (DXA) (Hologic QDR 4500; Hologic, Inc).^16^ The participants were directed to sit down in the middle of the scanning table and then lay supine with their spine aligned with the impressed longitudinal midline. Once they were in the supine position, their arms were placed along their side with the palms pronated to standardize the scanned area. A small gap was maintained between upper limbs and trunk (<5 cm) and between legs (<10 cm) to facilitate segmentation in regional assessments. LBM, fat body mass and bone mineral content were determined for the whole body and the following segments: legs, arms and trunk. Body composition variables in bilateral body regions (leg LBM and arm LBM) were computed as the sum of right and left limbs.

#### Cardiac structure, stiffness, function and haemodynamics

Apical four‐chamber and two‐chamber cine‐loops were recorded via high‐resolution ultrasound (Mindray Medical M9) (i) at rest and (ii) repeatedly throughout an established incremental exercise test (detailed in the subsequent ‘Aerobic capacity’ section) in a cycle ergometer (KICKR Core, Wahoo) setup designed to facilitate maximal effort and precise echocardiography, as previously described.^2^, ^3^, ^17^ Following the American Society of Echocardiography and the European Association of Cardiovascular Imaging recommendations, cardiac chamber quantification was performed offline using the modified Simpson method (biplane method of disks) by tracing the endocardial border of the LV in both apical four‐chamber and two‐chamber views at end‐diastole and end‐systole.^18^, ^19^ Stroke volume (SV) was determined as left ventricular end‐diastolic volume (LVEDV) minus LV end‐systolic volume (LVESV), while the product of SV and heart rate (HR) provided cardiac output (Q). The recommended Cube algorithm was used to quantify LV_mass_.^18^ LV relative wall thickness (LVRWT) was determined according to the equation: LVRWT = 2 × LV posterior wall thickness at end‐diastole (LVPWd)/LV internal diameter at end‐diastole (LVIDd). With respect to cardiac stiffness, LV end‐systolic elastance (Ees = systolic blood pressure (SBP) /LVESV), LV diastolic elastance (Ed = E/e′/LV SV) and effective arterial elastance (Ea = SBP/LV SV) were determined conforming to consensus recommendations.^20^, ^21^ Right (RA) and left atrial (LA) volumes were measured using the single plane Simpson's method of disks from the apical four‐chamber view at end‐systole.^18^, ^22^ Right ventricular (RV) function was assessed via tissue Doppler imaging (TDI) of tricuspid annular (TA) velocities and plane systolic excursion (TAPSE).^18^ RV dimensions were not analysed due to the uncertain imaging quality of such a morphologically complex chamber.^23^ Regarding the assessment of LV function, transmitral inflow velocities were recorded via pulsed‐wave Doppler, with the sample volume placed between the mitral leaflet tips in the apical four‐chamber view. The peak inflow velocities during early (E) and late (A) diastole and the ratio (E/A) was calculated. Likewise, myocardial tissue e′ and a′ velocities were recorded via TDI and the E/e′ ratio was calculated.^18^ Furthermore, the myocardial performance index (MPI), a global marker of LV diastolic and systolic function, was calculated as the sum of isovolumic relaxation and contraction times divided by ejection time.^18^ Blood pressures, comprising SBP, diastolic (DBP) and mean arterial pressure (MAP), were assessed at rest in the upper arm via the gold standard auscultatory method using a mercury sphygmomanometer. The ratio of MAP and Q yielded total peripheral resistance (TPR). The same method was successfully implemented during exercise to measure blood pressure and calculate TPR in a subset of women (*n* = 45) and men (*n* = 39) matched by age (40.4 ± 18.6 vs. 43.4 ± 18.7 year, *P* = 0.392) and physical activity (total MVPA: 5.6 ± 3.4 vs. 6.0 ± 3.8 hr · wk^−1^, *P* = 0.558; MVPA‐endurance: 4.8 ± 3.3 vs. 5.1 ± 3.6 hr · wk^−1^, *P* = 0.631). These haemodynamic assessments (SBP_AT_, DBP_AT_, MAP_AT_ and TPR_AT_) were performed at a fixed relative high intensity [anaerobic threshold (AT)] as determined by the point in which respiratory exchange ratio (RER) reaches 0.85 during the incremental exercise test.^24^

#### Aerobic capacity

O_2_ uptake (VO_2_) and CO_2_ output were continuously recorded via a mixing chamber system (CardioCoach VO_2_, KORR Medical, USA) throughout the incremental exercise test. Following a warm‐up period at 10–30 W, the workload was progressively increased by 10–30 W increments every 50 s until exhaustion was reached in the recommended total duration of 7–10 min.^25^ Calibration of the gas analysers and the flowmeter was conducted prior to each test. Values were averaged over 15 s.^26^ The highest average value defined peak O_2_ uptake (VO_2peak_) provided that at least two of the following criteria were fulfilled: (i) plateau in O_2_ uptake despite increased workload, (ii) age‐ and body position‐predicted HR_peak_ +/− 10 b.p.m.^27^, ^28^ and/or (iii) RER > 1.^29^ The arterio‐venous O_2_ difference at peak exercise (a‐vO_2diffpeak_) was determined according to the Fick Principle (VO_2peak_ = Q_peak_ × a‐vO_2 diffpeak_). Furthermore, blood O_2_ carrying capacity, as represented by haemoglobin (Hb) concentration, was measured at supine rest in venous blood (1 mL) from the antecubital region (ABL80, Radiometer).

### Statistical analysis

Statistical analyses were conducted via SPSS 26.0. Data were tested for normal distribution with the Kolmogorov–Smirnov test and for homogeneity of variances with the Levene's test. Sample size was determined as twice that of precedent studies to ensure high statistical power (>95% power to detect significant relationships (*P* > 0.05) between LBM and Fick components with a two‐sided α of 5%).^2^, ^3^ Study variables were compared between women and men via independent sample *t* tests. Linear regression analyses were implemented to assess the relationship of total and regional (leg, arm, trunk) LBM with cardiac structure/function, central and peripheral haemodynamics, and Fick Principle components (Q_peak_, VO_2peak_ and a‐vO_2diffpeak_) in each sex. The possible presence of non‐linearity was investigated via the residual versus fitted values plot. Pearson's correlation coefficient (*r*) was determined without (direct model) or with adjustment by potential moderating/confounding factors including (i) total or region‐specific fat percentage, to control for the influence of adiposity, and (ii) established CV risk factors [age, smoking, MAP, body mass index (BMI) and family history of heart disease]. A two‐tailed *P*‐value < 0.05 was considered significant.

## Results

### General characteristics

**Figure 1:**
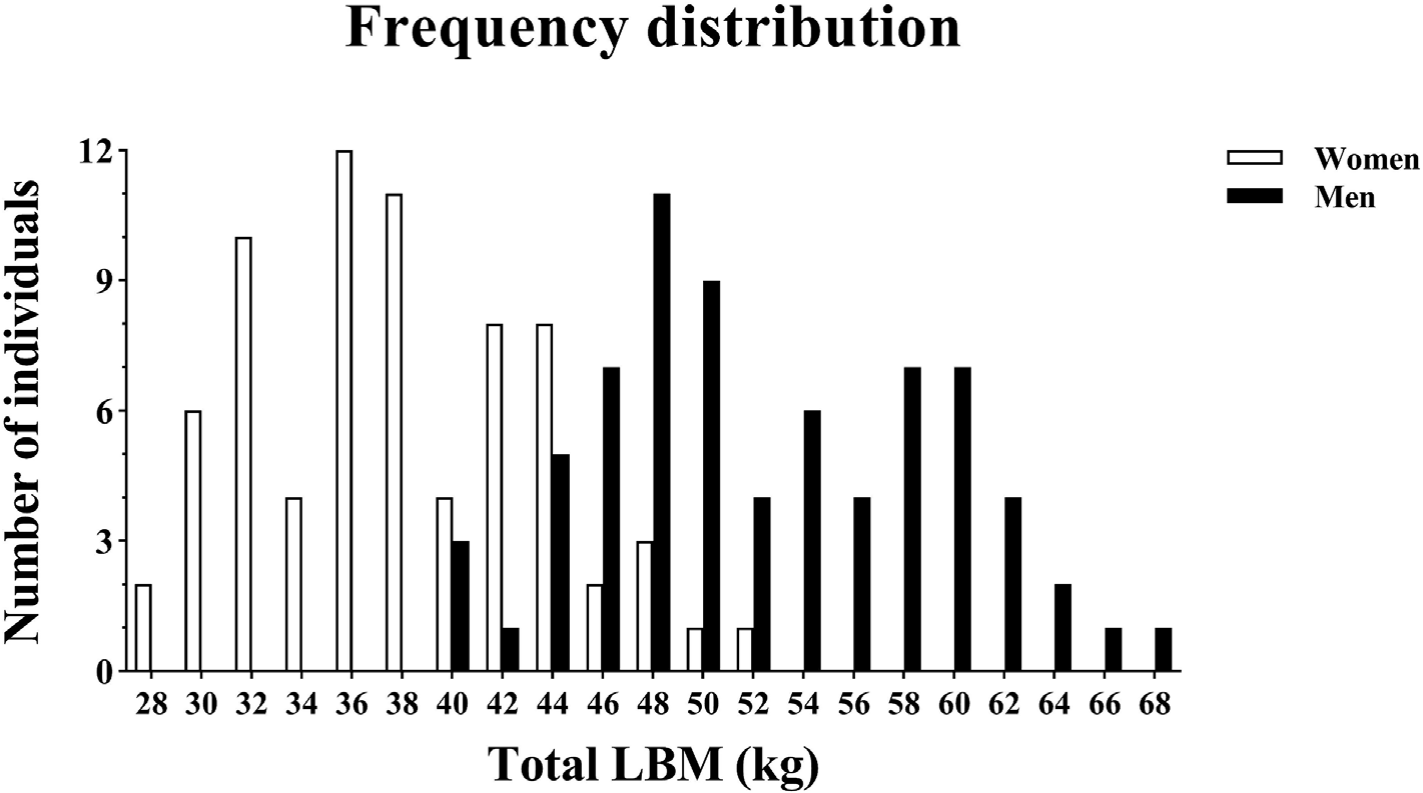
Frequency distribution of total lean body mass (LBM) in Hans Chinese (HC) women and men. LBM data (*X* axis) are grouped within intervals of equal width (2 kg).

Table 1 presents anthropometrics, body composition and resting cardiac variables in absolute units (i.e., not normalized by body size) in women and men. All individuals were non‐smokers and non‐obese (BMI < 30 kg · m^−2^). Approximately one‐third of women had reached menopause (*n* = 21), and none of them used hormone replacement therapy. The presence or absence of menopause did not alter the relationships found in the study. Age and physical activity (total and specific to endurance exercise) were matched between sexes (*P* ≥ 0.380). Women presented with smaller anthropometric indices (height, weight, BMI and body surface area) and lower blood O_2_ carrying capacity (Hb concentration) compared with men (*P* < 0.001). Regarding body composition, total and regional (leg, arm and trunk) LBM were reduced in women relative to men (*P* < 0.001), whereas the opposite was observed for total and regional fat percentage (*P* < 0.001). The histogram of LBM followed the normal curve frequency distribution in women and men, with substantial overlap between sexes (Figure 1, Figure S1). With respect to the cardiac phenotype at rest, women and men had a similar LV concentric/eccentric geometrical pattern (LVRWT, *P* = 0.117), although the internal dimensions of right and left chambers, wall thickness and LV_mass_ were smaller in women (*P* < 0.001). Resting cardiac systolic, diastolic and overall functions, as represented by TAPSE, mitral inflow/myocardial tissue velocities and myocardial performance index (MPI), did not differ between sexes (*P* ≥ 0.480).

### Relationship of lean body mass with left ventricular structure and stiffness

**Figure 2:**
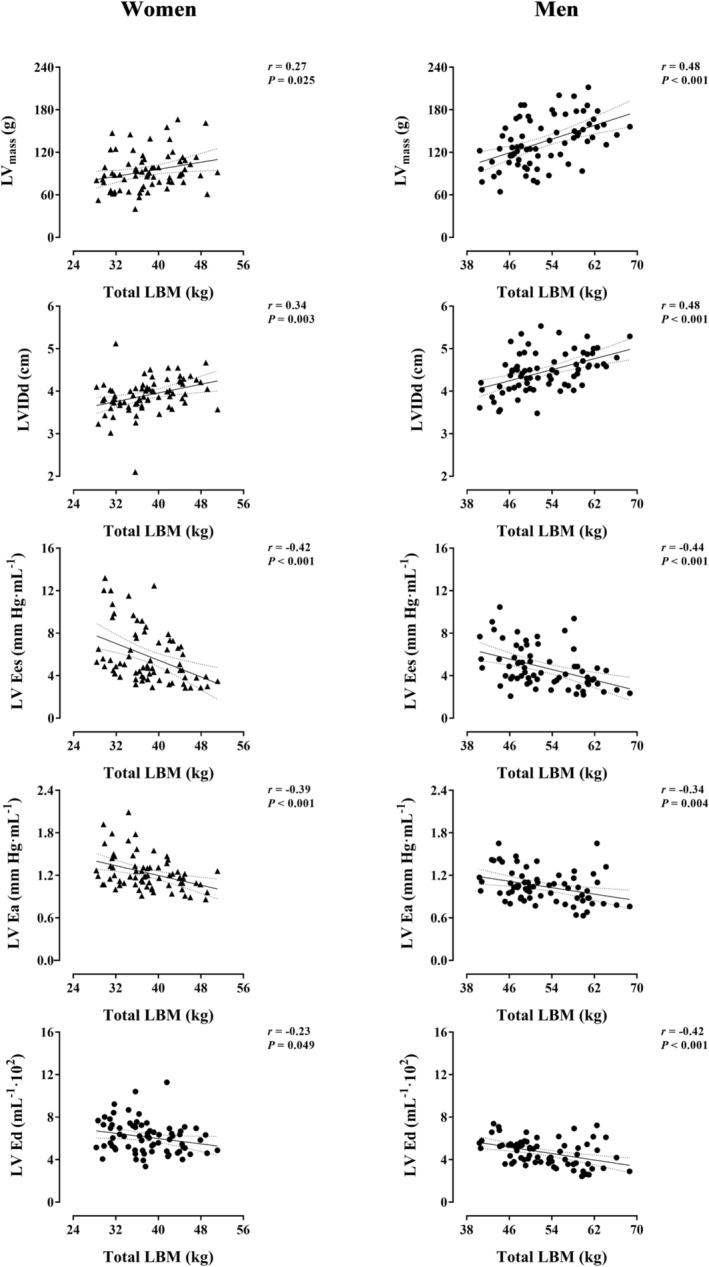
Relationship of total lean body mass (LBM) with left ventricular structure and stiffness in Hans Chinese (HC) women and men. Each graph includes the lines of best fit (95% confidence interval). Women and men are represented by triangles and circles, respectively. LV Ea, left ventricular‐arterial elastance; LV Ed, left ventricular diastolic elastance; LV Ees, left ventricular end‐systolic elastance; LVIDd, left ventricular internal diameter at end‐diastole; LV_mass_, left ventricular mass.

Figure 2 illustrates the main relationships of total LBM with LV structure and stiffness separated by sex. Total LBM positively associated with LV_mass_ and LVIDd in women and men (*r* ≥ 0.27, *P* ≤ 0.025). Moreover, total LBM exclusively and positively associated with LVPWd in men (*r* = 0.28, *P* = 0.016) (Table S1). As for LV and central arterial stiffness, total LBM negatively associated with LV Ees (*r* ≥ −0.42, *P* < 0.001), LV Ea (*r* ≥ −0.34, *P* ≤ 0.004) and LV Ed (*r* ≥ −0.23, *P* ≤ 0.049) in women and men (Figure 2). Similar relationships were found with the trunk instead of total LBM as the independent variable in both sexes (Table S1).

### Relationship of lean body mass with total peripheral resistance

Total LBM negatively associated with TPR in women and men (*r* ≥ −0.27, *P* ≤ 0.022) (Table S1). Likewise, regional (leg and arm) LBM negatively associated with TPR in women and men (*r* ≥ −0.24, *P* ≤ 0.048). During exercise, total and regional (leg, arm and trunk) LBM exclusively and negatively associated with TPR_AT_ in men (*r* ≥ 0.47, *P* ≤ 0.003) (Table S1).

### Relationship of lean body mass with left ventricular volumes at rest and peak exercise

**Figure 3:**
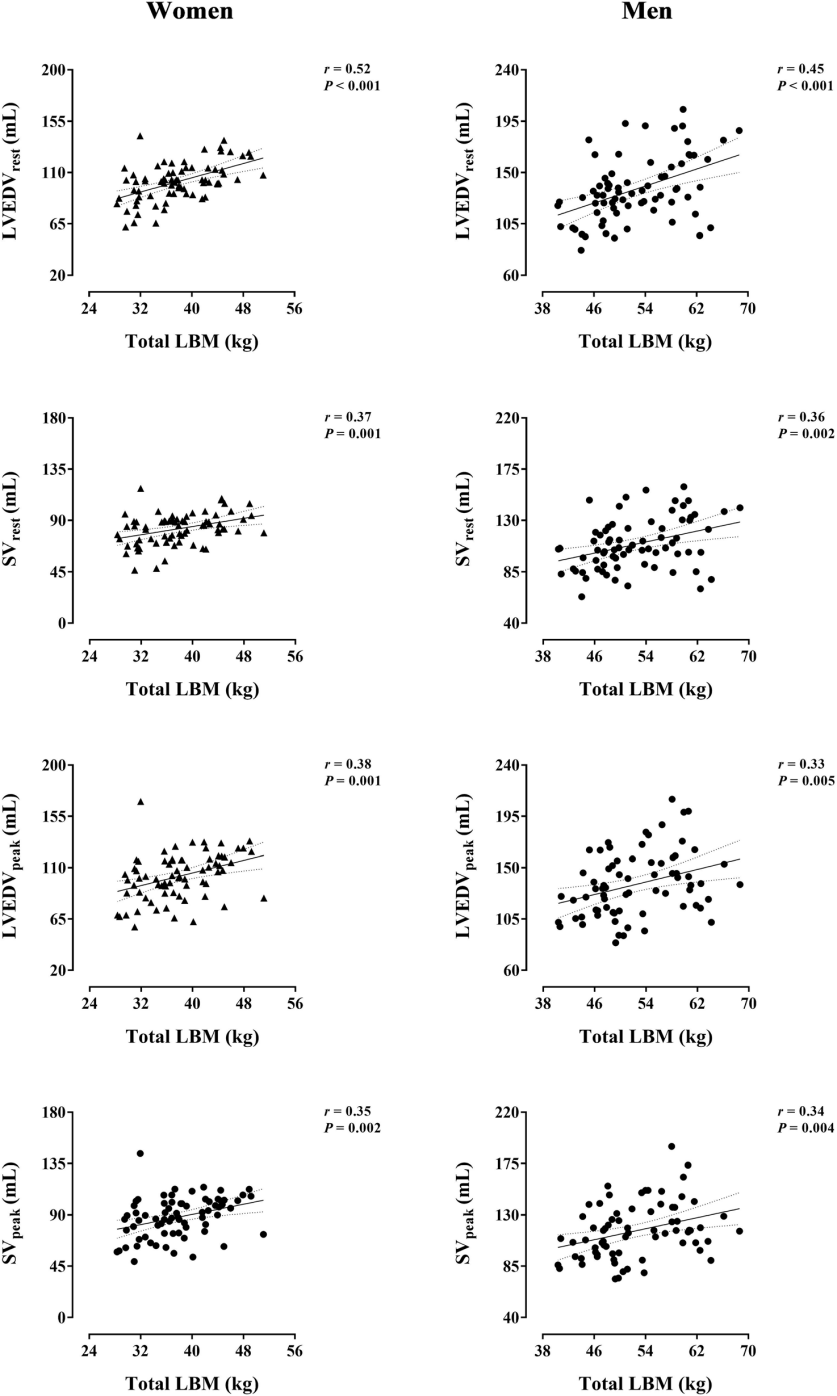
Relationship of total lean body mass (LBM) with left ventricular volumes at rest and peak exercise in Hans Chinese (HC) women and men. Each graph includes the lines of best fit (95% confidence interval). Women and men are represented by triangles and circles, respectively. LVEDV_peak_, left ventricular end‐diastolic volume at peak exercise; LVEDV_rest_, left ventricular end‐diastolic volume at supine rest; SV_peak_, left ventricular stroke volume at peak exercise. SV_rest_, left ventricular stroke volume at supine rest.

The relationship of total LBM with major LV volumes is presented separately by sex in Figure 3. Total LBM positively associated with LVEDV and SV at rest (*r* ≥ 0.36, *P* ≤ 0.002) and peak exercise (LVEDV_peak_, SV_peak_) (*r* ≥ 0.33, *P* ≤ 0.05) in women and men. Similar relationships were detected with regional LBM components (leg, arm and trunk) instead of total LBM in both sexes (Table S1). In addition, total and regional LBM positively associated with LVESV_peak_ in women (*r* ≥ 0.31, *P* ≤ 0.009), but not in men (*P* ≥ 0.066) (Table S1).

### Relationship of lean body mass with the Fick principle components

**Figure 4:**
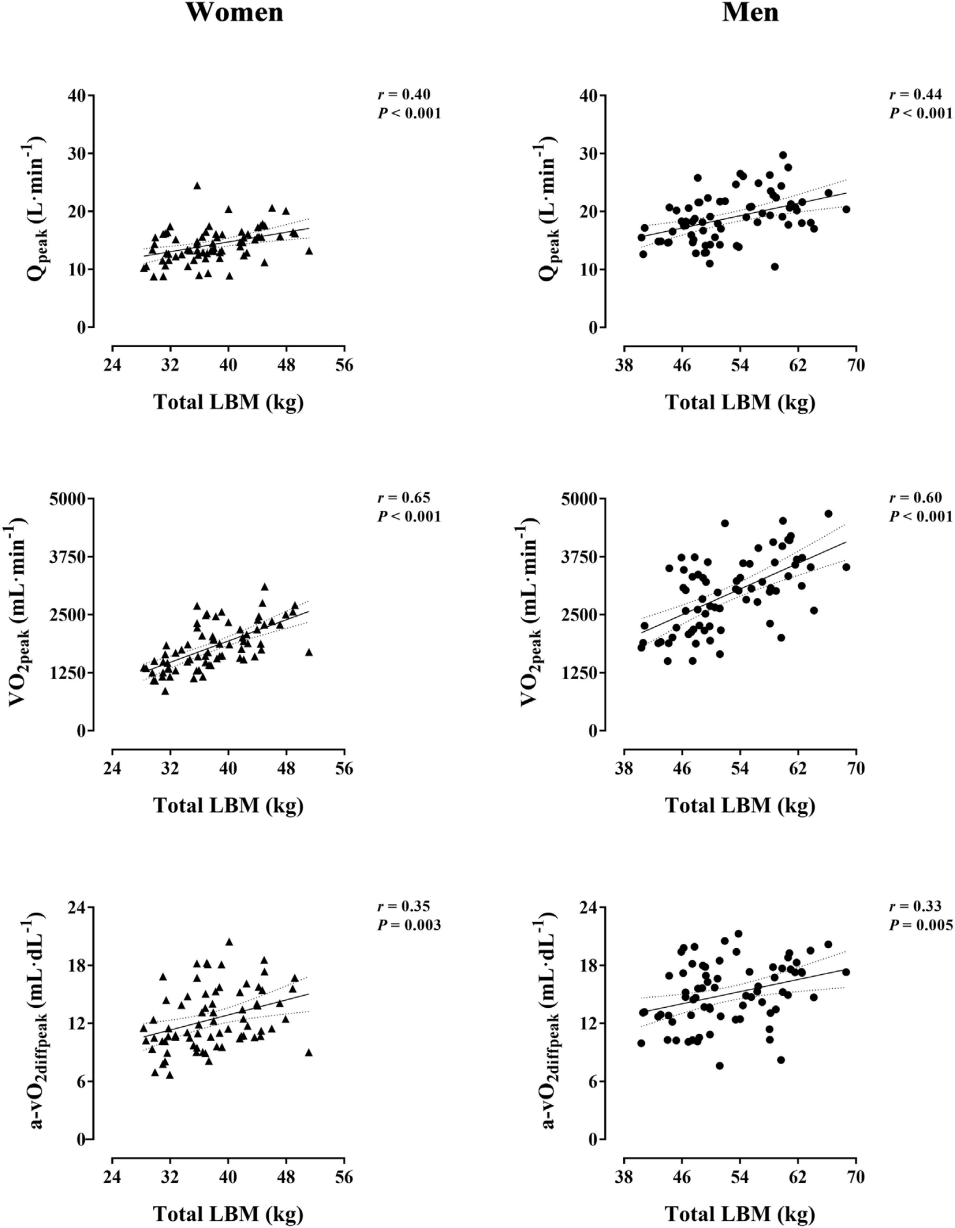
Relationship of total lean body mass (LBM) with the Fick principle components in Hans Chinese (HC) women and men. Each graph includes the lines of best fit (95% confidence interval). Women and men are represented by triangles and circles, respectively. A‐vO_2diffpeak_, arteriovenous O_2_ difference at peak exercise; Q_peak_, cardiac output at peak exercise; VO_2peak_, peak O_2_ consumption.

Figure 4 portrays the relationship of total LBM with the Fick Principle components separated by sex. Total LBM positively associated with Q_peak_, VO_2peak_ and a‐vO_2diffpeak_ in women and men (*r* ≥ 0.33, *P* ≤ 0.005). Among regional LBM, leg LBM prominently and positively associated with a‐vO_2diffpeak_ in women and men (*r* ≥ 0.43, *P* < 0.001) (Table S1).

### Influence of potential moderating and/or confounding factors on regression analyses

Covariate‐adjusted regression analyses were performed to assess the influence of adiposity and established CV risk factors. Adjustment by body fat percentage did not modify the significance status of the relationships between total LBM and study variables, except for LV Ed in women (*P* = 0.116) (Table S2). Relationships between total LBM and LV_mass_ were strengthened when adjusted by CV risk factors in both sexes (*r* ≥ 0.40, *P* < 0.001) (Table S3). The remaining relationships of total LBM were preserved, except for those with LV Ed, TPR and Q in women (*P* ≥ 0.066) and LV Ea in men (*P* = 0.289) (Table S3). Among regional LBM, leg LBM generally maintained, to a higher extent than arm or trunk LBM, its relationship with study variables after adjustment by region‐specific fat percentage and CV risk factors in women and men (Table S2 and Table S3).

## Discussion

This study determined the relationship of total and regional LBM with the resting and stressed cardiorespiratory phenotype of HC women and men matched by age and physical activity. The main general observations denote that total LBM is: (i) positively associated with the internal size and mass of the principal pumping chamber (LV); (ii) negatively associated with the stiffness of the LV and central arteries; (iii) positively associated with peak exercise LV volumes and Fick principle components (Q_peak_, VO_2peak_ and a‐vO_2diffpeak_). These relationships were present in both sexes and were prevailingly independent of (or strengthened by) the influence of adiposity and established CV risk factors.

Whether LBM and the CV system constitute an ethnic‐specific relationship compelled the current investigation. The principal discovery is the overarching relationship of LBM with fundamental cardiac and aerobic variables in HC men, contrasting with previously reported nonexistent LBM correlates in EA men with similar health and physical activity characteristics.^2^, ^3^ Such a marked divergence could be explained by ethnic‐specific quantitative attributes of body composition. In agreement with long‐standing findings,^11^ HC women and men presented with substantially less LBM than EA counterparts.^2^, ^3^ The HC‐EA ethnic gap was manifestly wider within the male sex, with ∼10 kg less of LBM on average in HC versus EA men.^2^, ^3^ Indeed, the average LBM in HC men fell within the female LBM range in EA.^2^, ^3^ Moreover, in comparison with EA,^2^, ^3^ a threefold greater overlap in LBM was observed between HC women and men, as displayed in Figure 1. Hence, unlike the clear prominence of LBM in EA men, HC men exhibit a rather female‐like LBM. Consequently, LBM may not be prevalently in *excess* (conforming to our previous terminology) in men from HC ethnicity.^2^, ^3^, ^6^ Mechanistically, the CV capacity to circulate blood and deliver O_2_ may be limited by low LBM as a result of restricted skeletal muscle‐induced arteriolar vasodilation and augmented resistance to blood flow (TPR), in HC men, *ad modum* EA women.^3^, ^6^, ^30^, ^31^ In fact, LBM negative associated with TPR at rest and during exercise in HC men. Moreover, LBM positively associated with LV myocardial thickness and HR_peak_ in men (Table S1), suggesting a male‐specific link between LBM and central (cardiac) mechanical potential to eject blood. Notwithstanding, further studies are needed to ascertain whether ethnic‐specific factors other than low or high LBM independently contribute to its association (or marked dissociation) with the CV system in the worldwide male population.

Previous studies have not investigated or reported the relationship of LBM with the stiffness of the heart. The loss of cardiac compliance is a pathophysiological hallmark leading to and expediting CV dysfunction and morbidity.^21^, ^32^, ^33^ Herein, LBM negatively associated with LV stiffness in the two phases of the cardiac cycle, diastole (LV Ed) and systole (LV Ees), in HC women and men (Figure 2). Notably, the relationship of LBM with LV Ees was preserved after adjustment by the moderating/confounding influence of adiposity and CV risk factors in both sexes (Table S2 and Table S3), diverging from the ‘adipocentric’ paradigm.^34^ Indirect mechanisms might in theory explain such a relationship. LV Ees reflects the stiffness of the myocardium at maximal cardiomyocyte activation, when the tension is highest per unit of myocardial surface area; it is thus considered an index of LV contractility.^21^ Provided the reasonable assumption that LV contractility as measured *in vivo* is partly a function of TPR,^35^ the negative relationship of LBM with TPR (independent of CV risk factors in HC men but not in women) could, in theory, contribute to explain the coupling between LBM and LV Ees. Namely, the heart of individuals with low LBM, plausibly entailing low skeletal muscle mass and consequently limited capacity to reduce TPR,^3^, ^6^ might be chronically constrained to enhance its contractility (myocardial tension) in order to deliver a certain volume of blood to the periphery. Nonetheless, the data obtained in this study does not support this hypothesis. In unplanned exploratory analyses, TPR as a covariate did not alter the relationship of LBM with LV Ees in women or men, further reinforcing the robustness of their bond. It should also be noted that, in women, LBM was not associated TPR_AT_ (Table S1). The lower sample size of these analyses and/or a ceiling for TPR reduction during exercise in women with very low LBM may explain this dissociation. Furthermore, the accuracy of DXA to assess LBM may be attenuated in individuals very low LBM. At present, the possibility remains that a direct but yet to‐be‐conjectured mechanism causes LV Ees from LBM. In this regard, shared molecular mechanisms homeostatically regulating muscle mass and myocardial stiffness, for example, titin isoform switching, may be proposed for further investigation.^36^ Were a direct mechanistic link eventually established, increases in LBM alone would relieve LV stiffness (i.e., increasing LV distensibility), an appealing prospect to beneficially modify an age‐dependent and seemingly irreversible cardiac maladaptation expediting the development of heart failure with preserved ejection fraction, a prevalent disease with few therapeutic options available.^6^, ^32^

**Figure 5:**
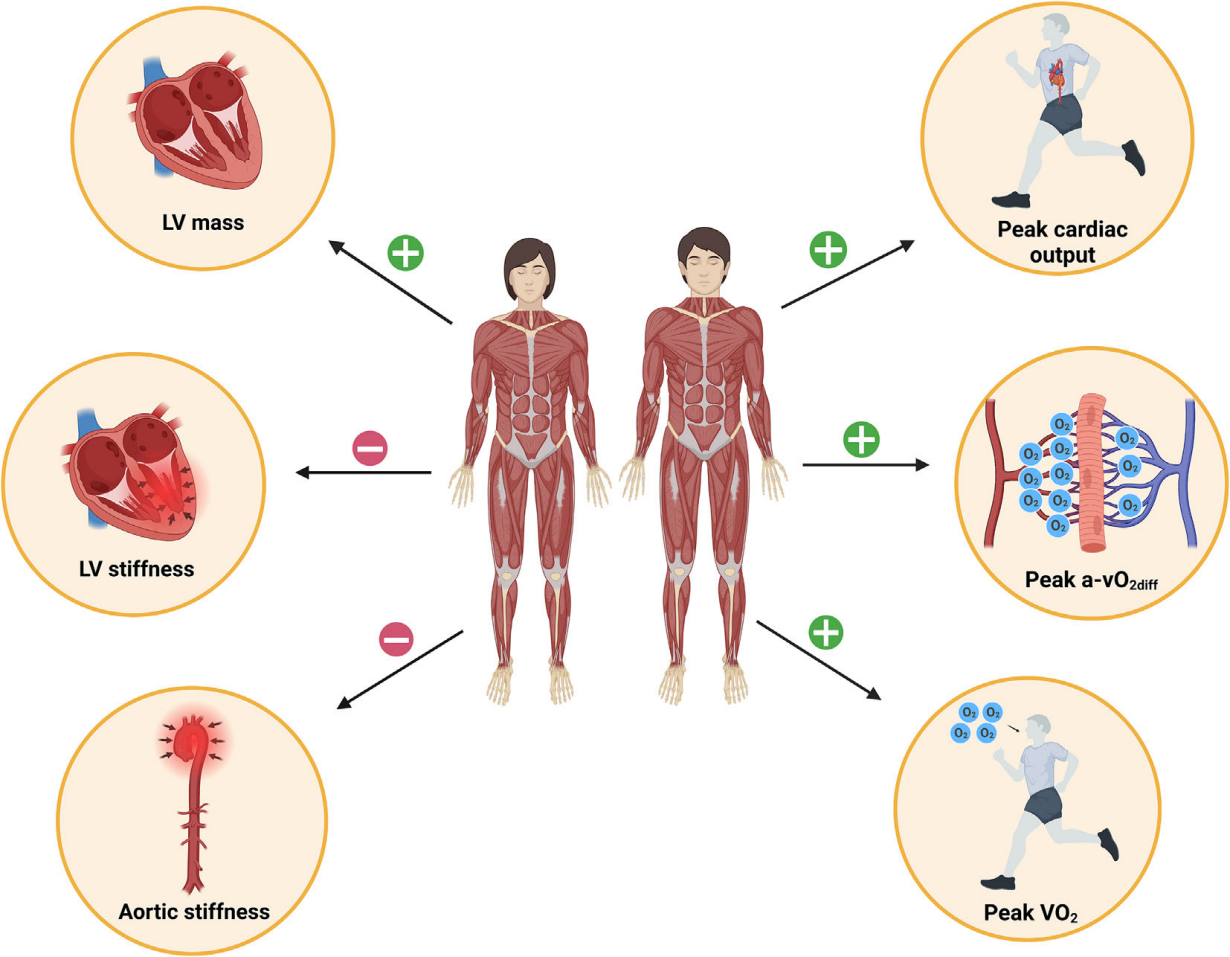
Illustrative synopsis of the relationship of total lean body mass (LBM) with the cardiac and aerobic phenotype in Hans Chinese (HC) women and men. A‐vO_2diff_, arteriovenous O_2_ difference; LV, left ventricle; VO_2_, O_2_ consumption.

Structural and functional attributes of the CV system coalesce in the inherently related capacities to deliver and consume O_2_. In healthy as well as in the vast majority of diseased individuals, the higher the cardiac pumping capacity (Q_peak_), the greater the O_2_ consumption (VO_2peak_), conforming to the Fick Principle.^5^, ^37^ Both Q_peak_ and VO_2peak_ are powerful surrogate endpoints for CV and all‐cause mortality as well as crucial outcomes in selecting candidates for heart transplantation.^4^, ^5^, ^38^, ^39^ Recently, the unexpectedly strong independent relationship of LBM with Q_peak_ and VO_2peak_ was unveiled in EA women, while the potential underlying mechanisms were exposed.^2^, ^3^, ^6^ These results are extended to HC women and men (Figure 4). Moreover, LBM positively associated with cardiac filling at peak exercise in both sexes (Figure 3), consistent with the forthright inference from the Frank–Starling mechanism—mainly established via animal experimentation and human investigations in EA populations.^37^ In other respects, no precedent study to our knowledge analysed the relationship of LBM with the remaining Fick Principle component: a‐vO_2diffpeak_, reflecting peak O_2_ extraction. The relationship identified between leg LBM and a‐vO_2diffpeak_ in HC women and men, which was preserved after adjustment by moderating/confounding factors, suggests an independent effect of muscle mass on O_2_ extraction in the exercising limbs (Figure 4, Table S1). This effect can be attributed to adaptations (increases) in leg LBM leading to (i) optimized systemic blood flow distribution redirecting a higher fraction of Q_peak_ towards the exercising lower limbs and/or (ii) intrinsically enhanced muscular capacity to extract and consume O_2_ from the circulation.^40^ Therefore, LBM not only may facilitate systemic O_2_ delivery but contribute to efficient regional O_2_ distribution and/or extraction in HC women and men (Figure 5). A prevailing and pronounced anthropometric trait in HC populations, that is, shorter lower limbs relative to body height, might partly underpin the role of leg LBM in the integrative capacity of CV and muscular systems to deliver and consume O_2_.^41^

### Limitations

Whether the present findings, obtained in the healthy population to limit the influence of moderating/confounding pathophysiological factors, can be extrapolated to clinical conditions with altered cardiovascular phenotypes and/or abnormal body composition remains to be elucidated. A substantial moderating effect of adiposity on the relationship of LBM with the cardiac phenotype might be expected in the presence of chronic inflammatory diseases.^34^ In other respects, human studies assessing sex differences typically have at their disposal limited tools to perfectly discern and control for constitutive (e.g., genetic), development and lifestyle factors. Potential studies in female/male dizygotic twins, which share ∼50% of DNA and plausibly numerous development‐ and lifestyle‐related factors, may reduce such a source of confounding. Finally, studies specifically measuring muscle mass, density, function and quality (e.g., assessing skeletal muscle mitochondrial content and oxidative capacity) will provide further insight. Notwithstanding, the results were additionally ascertained in body regions that are little affected by confounding methodological factors.^42^

## Conclusions

This study discloses a widespread relationship between LBM and the cardiac phenotype, including LV structure, stiffness and function in resting and peak exercise conditions in HC adults, irrespective of sex. Moreover, LBM strongly associates with aerobic capacity (VO_2peak_) and its Fick determinants (Q_peak_, a‐vO_2diffpeak_) in HC women and men. The predominantly independent nature of these associations from main moderating/confounding factors, suggests a potential intrinsic relationship between LBM and the functional capacity of the Chinese CV and aerobic energy systems. Future experimental studies may explore the previously unforeseen impact of LBM modification on CV and metabolic health in the world's largest ethnic group.

## Funding

This was funded by Research Grant Council of Hong Kong ‐ Early Career Scheme (106210224 to D.M.) and Seed Fund (104006024 to D.M.).

## Conflict of interest statement

The authors declare that they have no competing interests.

## Supporting information


**Table S1.** Relationship of lean body mass (LBM) with left ventricular structure/function, total peripheral resistance (TPR), cardiac and aerobic capacities in Hans Chinese (HC) women and men.
**Table S2.** Relationship of lean body mass (LBM) with left ventricular structure/function, total peripheral resistance (TPR), cardiac and aerobic capacities in Hans Chinese (HC) women and men, adjusted by body fat percentage.
**Table S3.** Relationship of lean body mass (LBM) with left ventricular structure/function, total peripheral resistance (TPR), cardiac and aerobic capacities in Hans Chinese (HC) women and men, adjusted by cardiovascular risk factors.
**Figure S1.** Frequency distribution of regional lean body mass (LBM) in Hans Chinese (HC) women and men.

## Data Availability

All data associated with this study will be available upon request to the corresponding author.
