# Supplementary material for: Lean body mass and the cardiorespiratory phenotype: An ethnic‐specific relationship in Hans Chinese women and men
Source: J Cachexia Sarcopenia Muscle. 2024 Apr 17;15(3):963–74. doi: 10.1002/jcsm.13464 (PMC11154775; doi:10.1002/jcsm.13464)
Supplement: Supplementary file 1 — Table S1. Relationship of lean body mass (LBM) with left ventricular structure/function, total peripheral resistance (TPR), cardiac and aerobic capacities in Hans Chinese (HC) women and men. Table S2. Relationship of lean body mass (LBM) with left ventricular structure/function, total peripheral resistance (TPR), cardiac and aerobic capacities in Hans Chinese (HC) women and men, adjusted by body fat percentage. Table S3. Relationship of lean body mass (LBM) with left ventricular structure/function, total peripheral resistance (TPR), cardiac and aerobic capacities in Hans Chinese (HC) women and men, adjusted by cardiovascular risk factors. Figure S1. Frequency distribution of regional lean body mass (LBM) in Hans Chinese (HC) women and men. [file JCSM-15-963-s001.docx]

**SUPPLEMENTAL MATERIAL**

- **Table S1.** Relationship of lean body mass (LBM) with left ventricular structure/function, total peripheral resistance (TPR), cardiac and aerobic capacities in Hans Chinese (HC) women and men.
- **Table S2.** Relationship of lean body mass (LBM) with left ventricular structure/function, total peripheral resistance (TPR), cardiac and aerobic capacities in Hans Chinese (HC) women and men, adjusted by body fat percentage.
- **Table S3.** Relationship of lean body mass (LBM) with left ventricular structure/function, total peripheral resistance (TPR), cardiac and aerobic capacities in Hans Chinese (HC) women and men, adjusted by cardiovascular risk factors.
- **Figure S1.** Frequency distribution of regional lean body mass (LBM) in Hans Chinese (HC) women and men.

**Table S1**. Relationship of lean body mass (LBM) with left ventricular structure/function, total peripheral resistance (TPR), cardiac and aerobic capacities in Hans Chinese (HC) women and men

|  | **Total LBM** | | | | **Leg LBM** | | | | **Arm LBM** | | | | **Trunk LBM** | | | |
| --- | --- | --- | --- | --- | --- | --- | --- | --- | --- | --- | --- | --- | --- | --- | --- | --- |
|  | **Women** | | **Men** | | **Women** | | **Men** | | **Women** | | **Men** | | **Women** | | **Men** | |
|  | ***r*** | ***P*** | ***r*** | ***P*** | ***r*** | ***P*** | ***r*** | ***P*** | ***r*** | ***P*** | ***r*** | ***P*** | ***r*** | ***P*** | ***r*** | ***P*** |
| LV_mass_ | 0.265 | **0.025** | 0.483 | **<0.001** | 0.149 | 0.211 | 0.456 | **<0.001** | 0.264 | **0.025** | 0.460 | **<0.001** | 0.352 | **0.002** | 0.463 | **<0.001** |
| LVIDd | 0.342 | **0.003** | 0.481 | **<0.001** | 0.289 | **0.014** | 0.526 | **<0.001** | 0.288 | **0.014** | 0.410 | **<0.001** | 0.366 | **0.002** | 0.418 | **<0.001** |
| LVPWd | 0.058 | 0.628 | 0.282 | **0.016** | -0.012 | 0.921 | 0.220 | 0.064 | 0.090 | 0.455 | 0.296 | **0.012** | 0.117 | 0.326 | 0.307 | **0.009** |
| LV Ees | -0.418 | **<0.001** | -0.442 | **<0.001** | -0.481 | **<0.001** | -0.517 | **<0.001** | -0.368 | **0.002** | -0.367 | **0.002** | -0.335 | **0.005** | -0.371 | **0.002** |
| LV Ed | -0.232 | **0.049** | -0.417 | **<0.001** | -0.201 | 0.091 | -0.407 | **<0.001** | -0.247 | **0.037** | -0.284 | **0.016** | -0.245 | **0.038** | -0.413 | **<0.001** |
| LV Ea | -0.393 | **<0.001** | -0.342 | **0.004** | -0.410 | **<0.001** | -0.377 | **0.002** | -0.330 | **0.006** | -0.278 | **0.022** | -0.344 | **0.004** | -0.286 | **0.018** |
| TPR | -0.273 | **0.022** | -0.370 | **0.002** | -0.270 | **0.024** | -0.327 | **0.007** | -0.237 | **0.048** | -0.340 | **0.005** | -0.234 | 0.051 | -0.368 | **0.002** |
| TPR_AT_ | -0.238 | 0.115 | -0.537 | **<0.001** | -0.280 | 0.063 | -0.589 | **<0.001** | -0.272 | 0.070 | -0.465 | **0.003** | -0.172 | 0.260 | -0.469 | **0.003** |
| LVEDV | 0.523 | **<0.001** | 0.451 | **<0.001** | 0.477 | **<0.001** | 0.473 | **<0.001** | 0.530 | **<0.001** | 0.349 | **0.003** | 0.506 | **<0.001** | 0.412 | **<0.001** |
| LVEDV_peak_ | 0.377 | **0.001** | 0.331 | **0.005** | 0.322 | **0.006** | 0.311 | **0.008** | 0.320 | **0.006** | 0.301 | **0.010** | 0.398 | **<0.001** | 0.331 | **0.004** |
| LVESV | 0.523 | **<0.001** | 0.508 | **<0.001** | 0.582 | **<0.001** | 0.560 | **<0.001** | 0.477 | **<0.001** | 0.418 | **<0.001** | 0.443 | **<0.001** | 0.453 | **<0.001** |
| LVESV_peak_ | 0.364 | **0.002** | 0.187 | 0.116 | 0.331 | **0.005** | 0.178 | 0.135 | 0.305 | **0.009** | 0.125 | 0.296 | 0.362 | **0.002** | 0.218 | 0.066 |
| SV | 0.373 | **0.001** | 0.363 | **0.002** | 0.294 | **0.012** | 0.370 | **0.001** | 0.407 | **<0.001** | 0.271 | **0.021** | 0.389 | **<0.001** | 0.337 | **0.004** |
| SV_peak_ | 0.352 | **0.002** | 0.338 | **0.004** | 0.297 | **0.011** | 0.316 | **0.007** | 0.298 | **0.011** | 0.318 | **0.006** | 0.377 | **0.001** | 0.332 | **0.004** |
| Q | 0.266 | **0.024** | 0.297 | **0.011** | 0.203 | 0.087 | 0.212 | 0.074 | 0.262 | **0.026** | 0.252 | **0.033** | 0.270 | **0.022** | 0.344 | **0.003** |
| Q_peak_ | 0.400 | **<0.001** | 0.437 | **<0.001** | 0.444 | **<0.001** | 0.431 | **<0.001** | 0.327 | **0.005** | 0.411 | **<0.001** | 0.359 | **0.002** | 0.429 | **<0.001** |
| HR_peak_ | -0.004 | 0.974 | 0.313 | **0.007** | 0.138 | 0.249 | 0.308 | **0.008** | -0.023 | 0.848 | 0.278 | **0.018** | -0.113 | 0.347 | 0.315 | **0.007** |
| VO_2peak_ | 0.650 | **<0.001** | 0.596 | **<0.001** | 0.744 | **<0.001** | 0.670 | **<0.001** | 0.519 | **<0.001** | 0.494 | **<0.001** | 0.554 | **<0.001** | 0.510 | **<0.001** |
| VO_2peak_/W_peak_ | 0.057 | 0.632 | 0.030 | 0.800 | 0.113 | 0.346 | 0.058 | 0.629 | -0.097 | 0.417 | -0.099 | 0.407 | 0.012 | 0.919 | -0.014 | 0.910 |
| a-vO_2diffpeak_ | 0.346 | **0.003** | 0.331 | **0.005** | 0.433 | **<0.001** | 0.446 | **<0.001** | 0.231 | 0.051 | 0.241 | **0.041** | 0.271 | **0.021** | 0.233 | **0.049** |

Significant associations (*P* < 0.05) are highlighted in bold.

a-vO_2diffpeak_, arteriovenous O_2_ difference at peak exercise; HR_peak_, peak heart rate; LBM, lean body mass; LV Ea, left ventricular-arterial elastance; LV Ed, left ventricular diastolic elastance; LVEDV, left ventricular end-diastolic volume; LVEDV_peak_, left ventricular end-diastolic volume at peak exercise; LV Ees, left ventricular end-systolic elastance; LVESV, left ventricular end-systolic volume; LVESV_peak_, left-ventricular end-systolic volume at peak exercise; LV_mass_, left ventricular mass; LVIDd, left ventricular internal diameter at end-diastole; LVPWd, left ventricular posterior wall thickness at end-diastole; Q, cardiac output; Q_peak_, peak cardiac output; *r*, Pearson correlation coefficient; SV, stroke volume; SV_peak_, stroke volume at peak exercise; TPR, total peripheral resistance; TPR_AT_, total peripheral resistance during exercise at the anaerobic threshold; VO_2peak_, peak oxygen consumption; W_peak_, peak workload.

**Table S2**. Relationship of lean body mass (LBM) with left ventricular structure/function, total peripheral resistance (TPR), cardiac and aerobic capacities in Hans Chinese (HC) women and men, adjusted by body fat percentage^1^

|  | **Total LBM** | | | | **Leg LBM** | | | | **Arm LBM** | | | | **Trunk LBM** | | | |
| --- | --- | --- | --- | --- | --- | --- | --- | --- | --- | --- | --- | --- | --- | --- | --- | --- |
|  | **Women** | | **Men** | | **Women** | | **Men** | | **Women** | | **Men** | | **Women** | | **Men** | |
|  | ***r*** | ***P*** | ***r*** | ***P*** | ***r*** | ***P*** | ***r*** | ***P*** | ***r*** | ***P*** | ***r*** | ***P*** | ***r*** | ***P*** | ***r*** | ***P*** |
| LV_mass_ | 0.311 | **0.008** | 0.461 | **<0.001** | 0.172 | 0.151 | 0.408 | **<0.001** | 0.324 | **0.006** | 0.412 | **<0.001** | 0.386 | **<0.001** | 0.464 | **<0.001** |
| LVIDd | 0.324 | **0.006** | 0.456 | **<0.001** | 0.251 | **0.035** | 0.458 | **<0.001** | 0.266 | **0.025** | 0.295 | **0.013** | 0.359 | **0.002** | 0.457 | **<0.001** |
| LVPWd | 0.115 | 0.339 | 0.265 | **0.025** | 0.063 | 0.601 | 0.178 | 0.138 | 0.170 | 0.157 | 0.253 | **0.033** | 0.147 | 0.220 | 0.306 | **0.009** |
| LV Ees | -0.296 | **0.014** | -0.416 | **<0.001** | -0.321 | **0.008** | -0.446 | **<0.001** | -0.177 | 0.148 | -0.228 | 0.063 | -0.299 | **0.013** | -0.426 | **<0.001** |
| LV Ed | -0.188 | 0.116 | -0.403 | **<0.001** | -0.089 | 0.458 | -0.400 | **<0.001** | -0.204 | 0.088 | -0.262 | **0.027** | -0.233 | 0.050 | -0.412 | **<0.001** |
| LV Ea | -0.318 | **0.008** | -0.312 | **0.010** | -0.321 | **0.008** | -0.340 | **0.005** | -0.234 | 0.055 | -0.225 | 0.067 | -0.311 | **0.010** | -0.288 | **0.018** |
| TPR | -0.244 | **0.044** | -0.371 | **0.002** | -0.268 | **0.026** | -0.342 | **0.005** | -0.208 | 0.086 | -0.341 | **0.005** | -0.213 | 0.079 | -0.367 | **0.002** |
| TPR_AT_ | -0.123 | 0.428 | -0.519 | **<0.001** | -0.118 | 0.444 | -0.519 | **<0.001** | -0.129 | 0.403 | -0.400 | **0.013** | -0.098 | 0.528 | -0.504 | **0.001** |
| LVEDV | 0.485 | **<0.001** | 0.432 | **<0.001** | 0.402 | **<0.001** | 0.448 | **<0.001** | 0.500 | **<0.001** | 0.311 | **0.008** | 0.491 | **<0.001** | 0.413 | **<0.001** |
| LVEDV_peak_ | 0.401 | **<0.001** | 0.334 | **0.004** | 0.311 | **0.008** | 0.312 | **0.008** | 0.385 | **<0.001** | 0.327 | **0.005** | 0.417 | **<0.001** | 0.332 | **0.005** |
| LVESV | 0.440 | **<0.001** | 0.484 | **<0.001** | 0.465 | **<0.001** | 0.507 | **<0.001** | 0.342 | **0.003** | 0.311 | **0.008** | 0.420 | **<0.001** | 0.489 | **<0.001** |
| LVESV_peak_ | 0.325 | **0.006** | 0.167 | 0.164 | 0.251 | **0.035** | 0.139 | 0.248 | 0.281 | **0.018** | 0.101 | 0.400 | 0.346 | **0.003** | 0.215 | 0.072 |
| SV | 0.371 | **0.001** | 0.358 | **0.002** | 0.254 | **0.032** | 0.364 | **0.002** | 0.433 | **<0.001** | 0.273 | **0.021** | 0.393 | **<0.001** | 0.336 | **0.004** |
| SV_peak_ | 0.389 | **<0.001** | 0.347 | **0.003** | 0.297 | **0.012** | 0.327 | **0.005** | 0.378 | **0.001** | 0.355 | **0.002** | 0.404 | **<0.001** | 0.334 | **0.004** |
| Q | 0.309 | **0.009** | 0.339 | **0.004** | 0.261 | **0.028** | 0.266 | **0.025** | 0.330 | **0.005** | 0.325 | **0.006** | 0.290 | **0.014** | 0.358 | **0.002** |
| Q_peak_ | 0.371 | **0.001** | 0.434 | **<0.001** | 0.382 | **<0.001** | 0.438 | **<0.001** | 0.306 | **0.009** | 0.417 | **<0.001** | 0.349 | **0.003** | 0.428 | **<0.001** |
| HR_peak_ | -0.094 | 0.437 | 0.292 | **0.013** | 0.043 | 0.722 | 0.302 | **0.011** | -0.143 | 0.235 | 0.227 | 0.057 | -0.164 | 0.172 | 0.315 | **0.008** |
| VO_2peak_ | 0.590 | **<0.001** | 0.611 | **<0.001** | 0.642 | **<0.001** | 0.622 | **<0.001** | 0.367 | **0.002** | 0.378 | **0.001** | 0.561 | **<0.001** | 0.600 | **<0.001** |
| a-vO_2diffpeak_ | 0.239 | **0.045** | 0.283 | **0.017** | 0.314 | **0.008** | 0.343 | **0.003** | 0.058 | 0.631 | 0.039 | 0.745 | 0.224 | 0.060 | 0.270 | **0.023** |

Significant associations (*P* < 0.05) are highlighted in bold.

^1^ All associations were adjusted by total or regional-specific body fat percentage, as applicable.

a-vO_2diffpeak_, arteriovenous O_2_ difference at peak exercise; HR_peak_, peak heart rate; LBM, lean body mass; LV Ea, left ventricular-arterial elastance; LV Ed, left ventricular diastolic elastance; LVEDV, left ventricular end-diastolic volume; LVEDV_peak_, left ventricular end-diastolic volume at peak exercise; LV Ees, left ventricular end-systolic elastance; LVESV, left ventricular end-systolic volume; LVESV_peak_, left-ventricular end-systolic volume at peak exercise; LV_mass_, left ventricular mass; LVIDd, left ventricular internal diameter at end-diastole; LVPWd, left ventricular posterior wall thickness at end-diastole; Q, cardiac output; Q_peak_, peak cardiac output; *r*, Pearson correlation coefficient; SV, stroke volume; SV_peak_, stroke volume at peak exercise; TPR, total peripheral resistance; TPR_AT_, total peripheral resistance during exercise at the anaerobic threshold; VO_2peak_, peak oxygen consumption.

**Table S3**. Relationship of lean body mass (LBM) with left ventricular structure/function, total peripheral resistance (TPR), cardiac and aerobic capacities in Hans Chinese (HC) women

and men, adjusted by cardiovascular risk factors^1^

|  | **Total LBM** | | | | **Leg LBM** | | | | **Arm LBM** | | | | **Trunk LBM** | | | |
| --- | --- | --- | --- | --- | --- | --- | --- | --- | --- | --- | --- | --- | --- | --- | --- | --- |
|  | **Women** | | **Men** | | **Women** | | **Men** | | **Women** | | **Men** | | **Women** | | **Men** | |
|  | ***r*** | ***P*** | ***r*** | ***P*** | ***r*** | ***P*** | ***r*** | ***P*** | ***r*** | ***P*** | ***r*** | ***P*** | ***r*** | ***P*** | ***r*** | ***P*** |
| LV_mass_ | 0.400 | **<0.001** | 0.518 | **<0.001** | 0.316 | **0.010** | 0.472 | **<0.001** | 0.330 | **0.007** | 0.450 | **<0.001** | 0.469 | **<0.001** | 0.496 | **<0.001** |
| LVIDd | 0.330 | **0.007** | 0.450 | **<0.001** | 0.278 | **0.024** | 0.451 | **<0.001** | 0.252 | **0.041** | 0.330 | **0.008** | 0.360 | **0.003** | 0.405 | **<0.001** |
| LVPWd | 0.106 | 0.399 | 0.353 | **0.004** | 0.053 | 0.673 | 0.277 | **0.027** | 0.114 | 0.362 | 0.334 | **0.007** | 0.155 | 0.215 | 0.385 | **0.002** |
| LV Ees | -0.380 | **0.002** | -0.272 | **0.029** | -0.306 | **0.013** | -0.325 | **0.009** | -0.407 | **<0.001** | -0.178 | 0.160 | -0.385 | **0.002** | -0.222 | 0.077 |
| LV Ed | -0.227 | 0.066 | -0.313 | **0.012** | -0.220 | 0.075 | -0.311 | **0.012** | -0.219 | 0.077 | -0.159 | 0.209 | -0.224 | 0.071 | -0.310 | **0.013** |
| LV Ea | -0.314 | **0.011** | -0.135 | 0.289 | -0.279 | **0.024** | -0.180 | 0.155 | -0.271 | **0.029** | -0.094 | 0.459 | -0.304 | **0.014** | -0.067 | 0.601 |
| TPR | -0.209 | 0.091 | -0.122 | 0.338 | -0.162 | 0.194 | -0.085 | 0.505 | -0.193 | 0.121 | -0.144 | 0.256 | -0.200 | 0.108 | -0.123 | 0.335 |
| TPR_AT_ | -0.086 | 0.593 | -0.428 | **0.010** | -0.007 | 0.967 | -0.447 | **0.007** | -0.228 | 0.151 | -0.330 | 0.053 | -0.099 | 0.539 | -0.375 | **0.027** |
| LVEDV | 0.423 | **<0.001** | 0.337 | **0.006** | 0.388 | **0.001** | 0.375 | **0.002** | 0.440 | **<0.001** | 0.225 | 0.074 | 0.396 | **0.001** | 0.280 | **0.025** |
| LVEDV_peak_ | 0.361 | **0.003** | 0.306 | **0.014** | 0.385 | **0.001** | 0.312 | **0.012** | 0.260 | **0.035** | 0.270 | **0.031** | 0.327 | **0.007** | 0.285 | **0.023** |
| LVESV | 0.383 | **0.002** | 0.383 | **0.002** | 0.372 | **0.002** | 0.413 | **<0.001** | 0.414 | **<0.001** | 0.265 | **0.034** | 0.352 | **0.004** | 0.347 | **0.005** |
| LVESV_peak_ | 0.348 | **0.004** | 0.131 | 0.303 | 0.335 | **0.006** | 0.127 | 0.319 | 0.253 | **0.040** | 0.062 | 0.626 | 0.329 | **0.007** | 0.175 | 0.167 |
| SV | 0.351 | **0.004** | 0.294 | **0.018** | 0.314 | **0.010** | 0.332 | **0.007** | 0.370 | **0.002** | 0.193 | 0.126 | 0.332 | **0.006** | 0.235 | 0.061 |
| SV_peak_ | 0.340 | **0.005** | 0.324 | **0.009** | 0.372 | **0.002** | 0.331 | **0.008** | 0.241 | 0.051 | 0.300 | **0.016** | 0.303 | **0.013** | 0.289 | **0.021** |
| Q | 0.162 | 0.194 | 0.186 | 0.141 | 0.130 | 0.298 | 0.136 | 0.283 | 0.165 | 0.186 | 0.170 | 0.178 | 0.138 | 0.270 | 0.213 | 0.090 |
| Q_peak_ | 0.313 | **0.011** | 0.282 | **0.024** | 0.402 | **<0.001** | 0.303 | **0.015** | 0.230 | 0.063 | 0.283 | **0.024** | 0.244 | **0.048** | 0.263 | **0.036** |
| HR_peak_ | -0.146 | 0.242 | 0.023 | 0.855 | -0.068 | 0.589 | 0.001 | 0.992 | -0.092 | 0.462 | 0.047 | 0.710 | -0.212 | 0.087 | 0.071 | 0.577 |
| VO_2peak_ | 0.578 | **<0.001** | 0.528 | **<0.001** | 0.635 | **<0.001** | 0.572 | **<0.001** | 0.448 | **<0.001** | 0.371 | **0.003** | 0.514 | **<0.001** | 0.442 | **<0.001** |
| a-vO_2diffpeak_ | 0.238 | 0.055 | 0.305 | **0.014** | 0.252 | **0.042** | 0.374 | **0.002** | 0.142 | 0.256 | 0.142 | 0.263 | 0.226 | 0.068 | 0.218 | 0.083 |

Significant associations (*P* < 0.05) are highlighted in bold.

^1^ All associations were adjusted by age, smoking, mean arterial pressure, body mass index and family history of heart disease.

a-vO_2diffpeak_, arteriovenous O_2_ difference at peak exercise; HR_peak_, peak heart rate; LBM, lean body mass; LV Ea, left ventricular-arterial elastance; LV Ed, left ventricular diastolic elastance; LVEDV, left ventricular end-diastolic volume; LVEDV_peak_, left ventricular end-diastolic volume at peak exercise; LV Ees, left ventricular end-systolic elastance; LVESV, left ventricular end-systolic volume; LVESV_peak_, left-ventricular end-systolic volume at peak exercise; LV_mass_, left ventricular mass; LVIDd, left ventricular internal diameter at end-diastole; LVPWd, left ventricular posterior wall thickness at end-diastole; Q, cardiac output; Q_peak_, peak cardiac output; *r*, Pearson correlation coefficient; SV, stroke volume; SV_peak_, stroke volume at peak exercise; TPR, total peripheral resistance; TPR_AT_, total peripheral resistance during exercise at the anaerobic threshold; VO_2peak_, peak oxygen consumption.

**Figure S1.** Frequency distribution of regional lean body mass (LBM) in Hans Chinese (HC) women and men.

Each bilateral body region (leg LBM, arm LBM) is presented as the sum of LBM in right and left limbs.

LBM data (X axis) are grouped into intervals of equal width (1 kg for leg and trunk LBM, 0.5 kg for arm LMB).
